# Assessing determinants of SARS-CoV-2 infection in a large older adult representative sample

**DOI:** 10.1093/eurpub/ckac129.410

**Published:** 2022-10-25

**Authors:** G Mosconi, C Stival, C Signorelli, A Amerio, L Cavalieri d'Oro, L Iacoviello, D Stuckler, A Zucchi, A Odone, S Gallus

**Affiliations:** 1 Department of Public Health, University of Pavia, Pavia, Italy; 2 Department of Environmental Health Sciences, IRCCS Mario Negri Institute for Pharmacologic Research, Milan, Italy; 3 School of Medicine, Vita-Salute San Raffaele University, Milan, Italy; 4 DINOGMI, University of Genoa, Genoa, Italy; 5 IRCCS San Martino Polyclinic Hospital, Genoa, Italy; 6 Brianza Health Protection Agency, Monza, Italy; 7 EPIMED, Insubria University, Varese, Italy; 8 Department of Epidemiology and Prevention, IRCCS Neuromed, Pozzilli, Italy; 9 Department of Social Sciences and Politics, Bocconi University, Milan, Italy; 10 Bergamo Health Protection Agency, Bergamo, Italy

## Abstract

Most COVID-19-related deaths occurred in older adults, however to date, evidence on determinants of SARS-CoV-2 infection in this population is limited and mostly based on case series without a comparison group. A telephone-based cross-sectional study was conducted in November 2020 on a representative sample of 4,400 people aged ≥65 years from the Italian region of Lombardy. We determined the prevalence of participants reporting a SARS-CoV-2 infection in the period between the onset of the pandemic and the time of the interview. To investigate the determinants of the infection, we estimated odds ratios (OR) and their corresponding 95% confidence intervals (CI) thorough unconditional multiple logistic models. We further evaluated if the infection was a determinant of a worsening in mental health wellbeing. Overall, 4.9% of participants reported a history of SARS-CoV-2 infection. No significant relationship between sex and infection was observed. SARS-CoV-2 infection was less frequently reported in subjects aged ≥70 (OR = 0.55; 95% 0.41-0.74) compared to 65-69 years. We didn't observe any trend after 70 years of age. Participants reporting at least one chronic condition had a lower infection rate compared to healthy subjects (OR = 0.68 95% CI: 0.49-0.93). Separated/divorced subjects more frequently reported infection than married/cohabiting ones (OR = 2.33 95% CI: 1.29-4.20). Self-reported history of SARS-CoV-2 infection resulted being a determinant of an increase in depressive symptoms (OR = 1.57; 95% CI: 1.17-2.10). In this large study - among the few assessing the determinants of SARS-CoV-2 infection in a representative sample of older adults -, the prevalence of a history of infection in November 2020 approached 5%. We found that persons aged 70 and above and those with chronic conditions, thus individuals with likely less social interactions, were less frequently exposed to SARS-CoV-2 infection.

